# Temporal Stability of the Ruminal Bacterial Communities in Beef Steers

**DOI:** 10.1038/s41598-019-45995-2

**Published:** 2019-07-02

**Authors:** Brooke A. Clemmons, Cameron Martino, Liesel G. Schneider, Josh Lefler, Mallory M. Embree, Phillip R. Myer

**Affiliations:** 1 Department of Animal Science, University of Tennessee Institute of Agriculture, Knoxville, TN USA; 2 ASCUS Biosciences, Inc., San Diego, CA USA

**Keywords:** Applied microbiology, Microbiome

## Abstract

Nutritional studies involving ruminants have traditionally relied on relatively short transition or wash-out periods between dietary treatments, typically two to four weeks. However, little is known about adequate adaptation periods required to reach stabilization of the rumen microbiome that could provide more accurate results from nutritional studies in ruminants. This study determined the rumen bacterial communities and rumen environment parameters over ten weeks following transition from a forage-based to concentrate-based diet. Several α-diversity metrics, including observed OTUs and Simpson’s Evenness fluctuated throughout the trial, but were typically either greatest (observed OTUs) or lowest (Simpson’s) at week 5 of the trial contrasted from weeks 1 and 10 (*P* < 0.05). At week 4, several orders associated with the shift to the final bacterial community composition, including Pasteurellales, Aeromonadales, and Bacteroidales. At week 5, rumen pH was correlated with α-diversity (*P* = 0.005) and predictive of the rumen microbiome signature at week 10 (R^2^ = 0.48; *P* = 0.04). Rumen microbiome stability did not occur until approximately 9 weeks following adaptation to the diet and was associated with changes in specific bacterial populations and rumen environment. The results of this study suggest that adaptation and wash-out periods must be re-evaluated in order to accommodate necessary rumen microbiome acclimation.

## Introduction

The composition, structure, and function of the rumen microbiome in cattle is critical to the host’s health and nutrition, as these microbes are responsible for the breakdown of low-quality feedstuffs into energy substrates that can subsequently be utilized by the ruminant. Research continues to demonstrate that minor shifts in ruminal microbial taxa or abundances of specific microbes impacts livestock productivity. The rumen and gastrointestinal microbiomes in cattle have been linked to numerous aspects of host physiology and function, such as health^1^, nutrition^2^, feed efficiency^3^, and management^4^. Such research has been critical in understanding the role of the rumen microbiome in cattle production.

Due to the complex network of the rumen microbial community and its interaction with host fermentation and metabolism, diet stands as an important factor in ruminal microbiome composition and structure^5,6^. For example, cattle fed an exclusively forage-based diet have a distinct microbial profile from those on a high-grain diet, which is also reflected within core operational taxonomic units (OTUs)^7^. These outcomes are a direct result of the complexity of available substrates within the feed. Factors such as diet drive the day-to-day variation in microbiome stability; that is, maintenance of similar microbiota over time^8^. However, understanding the long-term variation in the ruminal microbiome is critical to maintaining ecological and functional equilibrium.

Microbiome stability can have significant implications for host physiology and health. For instance, stable microbiomes are associated with improved host immune response and overall host health^8,9^, whereas instability of microbiomes is associated with poor host health^9–11^. The microbiota, and in particular the gut microbiota, interact intimately with the host at a cellular level to impact host immune function^12,13^. Further, as described previously, the rumen microbiome performs vital functions for the host, and disruption of these functions by biotic or abiotic perturbations may influence the stability of the rumen microbiome and significantly alter animal health and production.

Thus far, the majority of research aimed at determining the influence of the microbiome on cattle production or the influence of cattle production on the ruminal microbiome has been conducted by examining short-term, end point sampling or single periods of sample collection. These single-point analyses have provided valuable insight to the influence of the rumen microbiome on livestock production but may confound the interpretation of study results and conclusions. Variation of the ruminal microbiota between animals is considerable^14,15^, and point samples may be not be satisfactory to adequately define an existing state within a population. Importantly, studies examining bovine nutrition with regard to the ruminal microbiome routinely rely on differences in diet or diet transitions, where the length of the study is defined by traditional nutritional parameters and historical data, not taking into account microbial acclimation to the study ration. The ruminal microbial temporal stability following such perturbations has yet to be determined. Thus, diet acclimation prior to experiments, diet re-acclimation among experimental periods, and other dietary changes in cattle gut microbiome studies may confound microbial characterization when temporal variation and stability are not taken into consideration. These patterns of variation may have significant implications when aiming to determine relationships between the gut microbiome and nutrition in cattle.

As microbial community stability following dietary changes may impact the length and results of *in vivo* research trials, as well as sampling frequency and interval, in this study, we examined the temporal diversity and stability of the ruminal bacterial communities in cattle following diet transition. The objective of this study was to determine bacterial community diversity and composition following diet transition and the duration required to achieve restoration of microbial stability.

## Results

### Sequencing information

A total number of 500 samples underwent microbial DNA extraction. Bacterial community composition was determined by amplifying and sequencing the V1–V3 hypervariable regions of the 16S rRNA gene. A total of 21,734,148 sequences remained following quality control and chimera removal. An average of 48,048 ± 41,628 sequences was present in each sample. After binning at 97% similarity, a total number of 21,401 OTUs were detected.

### Alpha diversity shifts over time

Alpha-diversity was measured using equitability, Simpson’s Evenness, chao1, and observed OTUs (Table 1). Good’s coverage was also measured to ensure satisfactory coverage of OTUs for each week (Table 1). Observed OTUs were greater during the first week (165.42 ± 4.24) compared to the final week (60.48 ± 4.00; *P* < 0.05, Table 1). Observed OTUs were greater during week 5 (307.61 ± 410), then decreased significantly in week 6 (45.60 ± 4.06; Table 1). Number of observed OTUs were greatest during week 5, followed by week 1 (Table 1). Equitability was greatest during week 1 of the trial (0.70 ± 0.02), fluctuated throughout the trial, but was lower at week 10 contrasted to week 1 (0.61 ± 0.02; *P* < 0.05). Whereas several metrics related to richness increased from week 1 to week 10, evenness fluctuated greatly (*P* < 0.05) but was similar by the end of the trial (0.11 ± 0.01) to the first week of the trial (0.13 ± 0.01; Table 1) as measured by Simpson’s Evenness (E). Simpson’s Evenness was lowest during week 5 (0.03 ± 0.01; Table 1).

**Table 1:** Alpha-diversity metrics of the rumen bacterial community for each week throughout the 70d trial.

Week	Observed OTUs	Chao1	Equitability	Simpson’s E	Good’s Coverage
1	165.42 ± 4.24^b^	166.88 ± 4.18^b^	0.70 ± 0.02^a^	0.11 ± 0.01^abc^	0.92 ± 0.04
2	110.33 ± 4.34^d^	111.14 ± 4.28^d^	0.59 ± 0.02^bc^	0.08 ± 0.01 ^cd^	0.94 ± 0.04
3	136.36 ± 4.24^c^	138.19 ± 4.18^c^	0.53 ± 0.02^c^	0.05 ± 0.01^de^	0.94 ± 0.04
4	89.25 ± 4.06^ef^	89.34 ± 4.00^ef^	0.61 ± 0.02^b^	0.09 ± 0.01^bc^	0.99 ± 0.00
5	307.61 ± 4.10^a^	322.38 ± 4.04^a^	0.53 ± 0.02^c^	0.03 ± 0.01^e^	0.98 ± 0.02
6	45.60 ± 4.06 ^h^	45.63 ± 4.00 ^h^	0.59 ± 0.02^bc^	0.14 ± 0.01^a^	0.99 ± 0.00
7	74.29 ± 4.06^fg^	74.38 ± 4.00^fg^	0.60 ± 0.02^b^	0.11 ± 0.01^abc^	0.99 ± 0.00
8	94.92 ± 4.06^de^	95.48 ± 4.00^de^	0.61 ± 0.02^b^	0.10 ± 0.01^bc^	0.99 ± 0.00
9	87.71 ± 4.15^ef^	87.88 ± 4.08^ef^	0.62 ± 0.02^b^	0.11 ± 0.01^abc^	0.96 ± 0.03
10	60.48 ± 4.06^gh^	60.66 ± 4.00^gh^	0.61 ± 0.02^b^	0.13 ± 0.01^ab^	0.99 ± 0.00

Data listed as least square means ± SEM. Differing superscripts within rows indicate significant differences (α ≤ 0.05) based on Tukey groupings.

### Bacterial community composition and rumen environment changes

**Figure 1:**
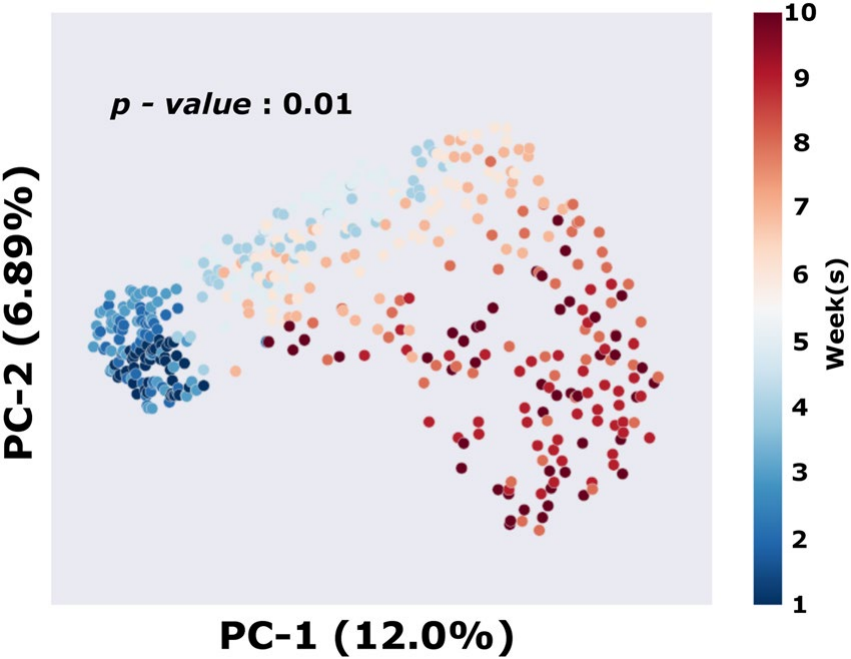
Principal coordinate analysis (PCoA) based on Bray-Curtis distances among the ruminal bacterial communities throughout the study (*P* = 0.01).

**Figure 2:**
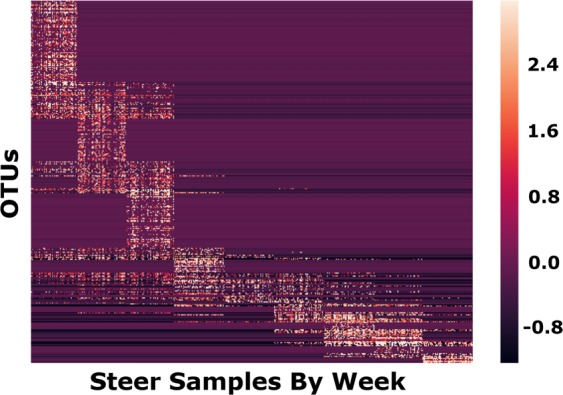
Spectral co-clustering of composite average of OTUs over time, indicating major microbial successions throughout the study in the rumen bacterial community.

**Figure 3:**
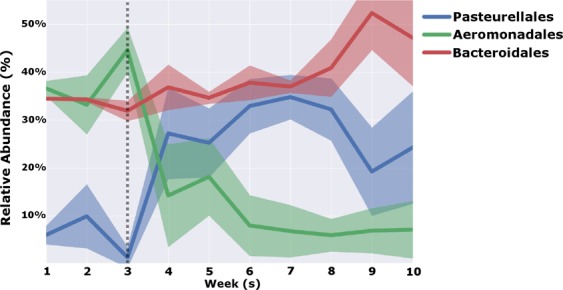
Relative abundance of three bacterial orders driving the shift in bacterial community composition throughout the 10-week trial. Shaded regions indicate SEM. The dotted line represents the timepoint at which shifts in bacterial orders began to occur.

The rumen bacterial communities began to shift at week 4 and reached stabilization by week 10, and the week 10 phylogenetic diversity differed from that of week 1 as indicated by PCoA (*P* = 0.01; Fig. 1). Decreased phylogenetic diversity was observed at week 1 compared to week 5 (Table 1); however, diversity decreased by week 10 (Table 1). Operational taxonomic unit abundances also changed following introduction of the new diet as illustrated using spectral co-clustering (Fig. 2). Three orders were identified through Random Forest feature selection as important temporal predictors, including Pasteurellales, Aeromonadales, and Bacteroidales. The shift to the final bacterial community composition began to occur at week 4 (Fig. 3).

**Figure 4:**
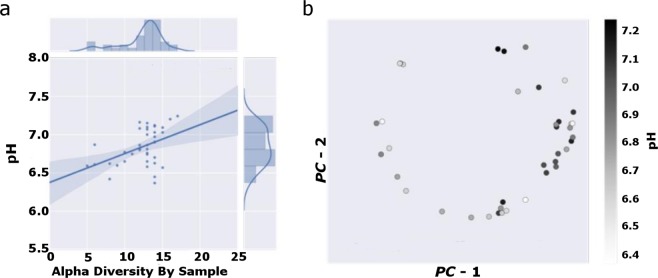
Rumen pH is an important factor in establishing bacterial community structure in week 5. (**a**) Rumen α-diversity correlated with pH (Pearson *R* = 0.44; *P* = 0.0051). (**b**) Rumen pH is predictive of bacterial community structure at week 5 (*R*^2^ = 0.48; *P* = 0.04).

Rumen environment changed as a result of diet change and/or variation in rumen bacterial community composition. Rumen pH was correlated with α-diversity at week 5 (*P* = 0.005; Fig. 4a). Rumen pH was also predictive of rumen microbiome signature at week 5 (R^2^ = 0.48; *P* = 0.04; Fig. 4b).

## Discussion

Beef cattle are a critical source of protein on a global scale, and of particular interest from both basic and applied research perspectives due to the unique nature of ruminants to convert inedible forages to high-quality protein and food products suitable for human consumption. The rumen microbiome produces an estimated 70% of the energy precursors required by the host beef cattle, predominantly due to the microbial conversion of low-quality feedstuffs to glucogenic precursors^16^. These glucogenic precursors, such as organic fatty acids including acetate and propionate, are subsequently absorbed by the animal. Nutritional studies involving beef cattle have historically relied on short adaptation and “wash-out” periods to diets or treatments. However, the research provided in this and other studies have demonstrated that short-duration dietary acclimation may not be sufficient time in order for the rumen microbiome to stabilize, which could influence the outcome of the trial, particularly as it relates the rumen microbiome and nutritional conclusions.

To reduce overestimation of OTUs, determine true diversity fluctuations, and characterize bacterial communities that may be driving bacterial shifts, machine learning methods were applied rather than traditional microbiome bioinformatics techniques. As expected, fewer OTUs were identified in this study than other studies in the rumen; however, several recent studies have suggested that the currently accepted number of OTUs reported are an overestimation as a result of amplification, sequencing, or other processing pipeline artifacts^17–20^. Furthermore, the use of supervised machine learning techniques, such as Random Forests, in addition to traditional analyses, allowed for the predictive assessment of ruminal bacterial community shifts during the transition from a pasture to a more rapidly-fermentable diet^21^. The application of supervised machine learning techniques to high-throughput sequencing data provides deeper interrogation of relationships among the host and its microbiota, and the relationships among the various members of the microbiome^22,23^.

In this study, the α-diversity of the rumen bacterial community was greatest at the start and middle of the trial, following a field-standard two-week adaptation period to the growing diet, and was greatly variable throughout much of the trial. Rumen bacterial community diversity was lower by the end of the trial, at ten weeks following the adaptation period. The rumen bacterial diversity was expectedly greatest at the start of the trial following the adaptation period, but reached stability by ten weeks. Other studies have established that diet is one of the greatest contributors to variation in the gastrointestinal microbiome^24–26^. The transition from a predominantly forage-based diet to a diet incorporating concentrates causes a shift in bacterial taxa due to changes in substrate type^27,28^. These differences in nutrient availability to the microbes may have resulted in a state of microbial community dysbiosis as bacterial populations competed for nutritional sources, increased functional redundancy occurred, and the physical environment of the rumen, such as pH, changed^29^.

Beyond intra-animal variation, diet is one of the greatest contributing factors to rumen microbial diversity^24^. Dietary transitions, adaptation periods, and wash-out periods are often incorporated into nutritional studies, including those involving the rumen microbiome^30^. These adaptations and wash-out periods have historically spanned anywhere from several days to four weeks, which previous studies have suggested is adequate time for acclimation, as reviewed by Brown *et al*.^31^ and supported by recent nutritional microbial studies^32^. However, in the current study, it took approximately ten weeks for the rumen bacterial community to stabilize; a duration much longer than traditionally utilized for adaptation or wash-out periods. Although cattle may physically acclimate to feed within a two-week period, the results of this study suggest the rumen microbiome requires additional time to stabilize.

Feature selection of taxonomic groups was used to identify those taxonomic groups that were important in explaining the change in microbiome over time, specifically the orders Aeromonadales, Pasteurellales, and Bacteroidales. At week 4 of this study, these three orders appeared to drive the shift to a stable bacterial community composition through a dramatic shift in relative abundance. Two of these orders, Aeromonadales and Pasteurellales, belong to the phylum Proteobacteria, and Bacteroidales belongs to the phylum Bacteroidetes. Proteobacteria and Bacteroidetes are typically two of the three most prevalent bacterial phyla found in the rumen, but bacteria belonging to the phylum Firmicutes are frequently found to be the most abundant in the rumen, followed by Bacteroidetes and Proteobacteria in cattle on a primarily forage-based diet^27,33,34^. However, as cattle transition to incorporate more readily-fermentable feedstuffs, Bacteroidetes becomes the dominant phylum^15,27^. This information may suggest that, although members of Proteobacteria are typically less abundant in the rumen overall, those microbes may play a larger role in the transition of the rumen microbiome from a forage to concentrate diet^27,35^. Proteobacteria are a large and greatly diverse phylum, further suggesting that low abundances of a variety of bacterial species may contribute significantly to divergences in host phenotypes in ruminants^36^.

The changes in abundances of these orders also provides additional insight given their function in the rumen. At week 4, relative abundance of Aeromonadales sharply decreased, whereas Bacteroidales and Pasteurellales both increased at week 4. Some rumen microbes belonging to Aeromonadales, including *Ruminobacter* and *Succinovibrio*, are fibrolytic in nature and found in conjunction with high-fiber diets^37,38^, which may account for the decrease in abundance of those microbes as the rumen microbiome adapted to the more readily digestible diet. Genera found in Bacteroidales and Pasteurellales, including *Prevotella* and *Actinobacillus*, respectively, are important for digestion of protein and carbohydrates^39–41^. Rapid production of byproducts of metabolism, such as organic acids, by Bacteroidales and Pasteurellales may be responsible for the shift in pH seen in week 5, which was also indicative of rumen microbiome signature^27,39,41^. The diet fed in this study included feedstuffs that are more readily fermentable^42^, which can cause decreases in rumen pH due to increased production of organic acids such as lactate^43^. This can shift the bacterial community composition towards those bacteria that are more tolerant of low pH, including those in Bacteroidales and Pasteurellales^27,44^.

This study analyzed the bacterial community composition stability following the transition from pasture to a more rapidly-fermentable diet. In a traditional cow-calf operation in the southeast region of the United States, calves are often placed on pasture prior to weaning with the dam^45,46^. At weaning, the calves are often placed on a backgrounding diet to improve the rate of gain before being sold to a feedlot^46,47^. This study analyzed the temporal stability of the rumen bacterial community composition in this type of system due to the importance and prevalence of this type of system in United States beef cattle production. Further studies could aim to determine the stability of the rumen microbiome during transition from a backgrounding diet to a feedlot diet that typically consists of predominantly grain^46,47^. Dietary transitions among different beef cattle production operations are important factors influencing microbiome function and stability, and supports the necessity to determine efficient microbial acclimation between diets in cattle production. For nutritional research, the opposite direction, in which cattle are transitioned from a predominantly grain or other rapidly-fermentable diet to a predominantly forage-based diet rarely occurs in beef enterprises; however, may provide valuable information regarding the time required to transition from a starch-efficient to a greater fiber-efficient microbiome, further defining factors affecting microbiome temporal stability.

Although previous studies have suggested that a two-week adaptation period may be adequate for nutritional studies in ruminants, in this study, the rumen bacterial community began to shift towards a stable bacterial community four weeks following the transition to the new diet and continued to stabilize in the subsequent five weeks. In nutritional studies involving cattle, a two-week adaptation and/or wash-out periods may not permit sufficient time for the rumen microbiome to stabilize. Rather, it is recommended based on this current study that adaptation or wash-out periods of at least eight weeks are incorporated for rumen microbiome and nutritional studies in order to ensure stability of the rumen microbiome. Instability of the rumen microbiome will likely influence nutritional and physiological production, and appropriately, study results. Thus, consideration should be given in the experimental design to account for ruminal microbial stability.

## Materials and Methods

This study was approved and carried out in accordance with the recommendations of the Institutional Animal Care and Use Committee at the University of Tennessee, Knoxville.

### Animal experimental design and sample collection

Fifty Angus steers of approximately seven months of age were maintained at the University of Tennessee Institute of Agriculture Plateau Research and Education Center in Crossville, TN, similarly to Clemmons *et al*.^42^. The steers weighed 264 ± 2.7 kg at the beginning of the trial. The steers grazed on mixed cool-season grasses until being transferred to the GrowSafe^©^ system (GrowSafe Systems Ltd., Airdrie, Canada) for a 14d adaptation period. Animals were subjected to a dietary step-up during the 14d adaptation period to transition to a growing diet (11.57% crude protein and 76.93% total digestible nutrients on a dry matter basis) consisting of 10% protein supplement, 10% cracked corn, and 80% corn silage with 28 mg monensin/kg DM. A 70d feed efficiency trial was administered following the acclimation period^42^. Weekly, rumen content samples were collected via gastric tubing^48^. Studies examining methods of oro-gastric tubing and cannulation sampling have demonstrated that oro-gastric tubing with collection of content and feed particles associated with the strainer, provides a representative rumen sample similar to a sample collected via the rumen cannula^49^. Approximately 100 mL of rumen content were transferred to 50 mL conical tubes, rumen content pH determined, frozen in an ethanol bath, and samples stored at −80 °C. Feed intake was continually monitored via the GrowSafe^©^ system throughout the 70d feed efficiency trial.

### DNA extraction and amplification

Rumen samples were centrifuged at 4,000 × g for 15 min, the supernatant was decanted and removed. Approximately 0.5 mL of resultant pellet was aliquoted for DNA extraction using the PowerViral® Environmental RNA/DNA Isolation Kit (Mo Bio Laboratories, Inc., Carlsbad, CA, USA). The 16S rRNA gene was amplified using 27 F^50^ and 534 R^51^ primers modified for Illumina sequencing, following standard protocols Q5® High-Fidelity DNA Polymerase (New England Biolabs, Inc., Ipswich, MA, USA)^52^. Following amplification, PCR products were verified with a standard 2% agarose gel electrophoresis and purified using AMPure XP bead (Beckman Coulter, Brea, CA, USA). The purified amplicon library was quantified and sequenced on the MiSeq Platform (Illumina, San Diego, CA, USA) according to standard protocols using a 2 × 300 v3, 600-cycle kit^53^. Raw fastq reads were de-multiplexed on the MiSeq Platform (Illumina, San Diego, CA, USA).

### Phylogenetic analysis

All raw sequencing data were trimmed of adapter sequences and phred33 quality filtered at a cutoff of 20 using Trim Galore^54^. All remaining sequences were then filtered for PhiX, low-complexity reads and cross-talk^55^. 16S taxonomic sequence clustering and classification was performed with the USEARCH’s UNOISE and SINTAX (v10.0.240)^56,57^ with the RDP 16S rRNA database v11.5^58^. Samples with less than 2,000 reads were removed from subsequent analysis due to low sequencing depth^59^.

### Statistical analyses

Downstream analysis was performed in python. PCoA was performed on Bray-Curtis distances and statistical significance was assessed through Analysis of Similarities (ANOSIM)^60^. Alpha diversity was measured both in dominance^61^ and singletons. Compositional normalization was performed through with the centered log-ratio transform using a pseudo count of one. Feature selection and supervised machine learning was performed on raw count data through Random Forests^62^, PCoA (Bray-Curtis) dimensionality reduction through scikit bio (http://scikit-bio.org/), data wrangling through pandas^63^, visualization through seaborn^64^ and matplotlib^65^, and machine learning and feature selection through scikit learn^66^.

Other measurements of α-diversity, including equitability, Simpson’s Evenness, Good’s coverage, and observed OTUs, were assessed for normality using SAS 9.4 using the PROC UNIVARIATE command (SAS Institute, Cary, NC). Outliers were detected and removed based on <10 observed OTUs, which included 15 total observations due to the biologically-improbable data. All variables followed a normal distribution based on visual observation of histogram and a Shapiro-Wilk score of ≥0.9. Data were analyzed using a mixed model ANOVA with random effect of steer and Tukey’s honest significant difference (HSD) post hoc test. Least square means with standard error and Tukey mean separation letter groupings indicating statistical differences by week are presented in Table 1.

## Data Availability

The datasets generated during and/or analyzed during the current study are available from the corresponding author(s) on reasonable request.
